# Supplementary material for: Are carbon emissions trading and green financial instruments synergistic? -Comprehensive quantitative research based on content analysis
Source: PLoS One. 2024 Mar 7;19(3):e0298601. doi: 10.1371/journal.pone.0298601 (PMC10919601; doi:10.1371/journal.pone.0298601)
Supplement: S1 Data — (ZIP) [file pone.0298601.s001.zip › Data/地级市Z_F工作报告.zip!/╣π╢1⁄2/╜¡├┼╩╨/└┤╫╘╣1⁄2╓┌║┼í╛┬φ┐╦╩2╛▌═°í┐_2017.docx]

各位代表：

我代表市政府向大会作政府工作报告，请予审议，并请政协各位委员和其他列席人员提出意见。

过去五年工作回顾

过去五年，市政府在省委、省政府和市委的正确领导下，在市人大及其常委会和市政协的监督支持下，狠抓“种树、搭桥修路、抓大项目”，围绕“兴业惠民、治吏简政”，全力打造“三门”[1]建设“三心”[2]。地区生产总值五年年均增长8.3%，成为全国小微企业创业创新基地示范城市、珠三角国家自主创新示范区、“中国制造2025”试点示范城市群的重要一员。特别是2016年，全市固定资产投资突破1500亿元，金融机构本外币贷款余额超2400亿元，均实现五年倍增；本外币存款余额突破4000亿元，一般公共预算收入突破200亿元，推动“放管服”[3]改革的做法得到李克强总理充分肯定，实现了第十四届政府工作良好收官、“十三五”规划顺利开局。

过去五年，是转型蓄势、谋局珠西的五年

我们不断深化江门发展思路，逐步强化“大江门”观念，凝聚各级各部门以及广大干部群众的力量，推动江门再出发。深化“三边”[4]战略，对外推动“东联西拓”[5]，主动对接广佛、深港澳两大龙头，对内推动“东提西进、同城共融”。牢牢把握发展实体经济这一根本，出台实施珠西行动、创新驱动等工作纲要及行动方案，制定并通过江门市国民经济和社会发展第十三个五年规划纲要和20个“十三五”重点专项规划。加快城市转型，目标由打造“三宜”（宜居宜游宜业）生态型城市提升为“四宜”（宜居宜游宜业宜创）的一流城市。推进文化名市建设，通过全国文明城市复评，立足侨、跳出侨，打造“中国侨都”城市名片。着力推进法治、透明、诚信、效率政府建设，提升政府“三为”（为企业、为群众、为基层）服务水平，营造良好政务环境。

我们抢抓作为“珠西战略”[6]策源地的机遇，全面落实省赋予的主战场、增长极新定位，推进产业结构转型升级。装备制造业占全市工业比重从2014年的24.7%提升到2016年的27.4%，工业技改投资年均增长93.5%，连续三年增速居全省前三位。珠三角（江门）国家自主创新示范区、广东（江门）大广海湾经济区、中欧（江门）中小企业国际合作区上升至国家战略层面，“1+6”核心园区[7]整合发展，江门产业转移工业园连续五年获省考评优秀等次，华夏幸福基业在华南地区布局的首个产业新城项目落户高新区。广东中车获动车组制造许可证，“江门制造”城际动车组交付使用，沈阳机床智能云制造华南总部落户。大力推进产业、交通、城建三大类项目建设，五年固定资产投资累计5800多亿元。实施精准招商，2014年以来全市共新引进投资额亿元以上装备制造业项目78个，计划总投资550.7亿元。

过去五年，是创新发展、经济提质的五年

我们大力实施创新驱动战略，在全国率先制定科技型小微企业认定标准、“小微双创”指数评价体系，出台实施“1+8”科技创新扶持政策[8]和“雏鹰计划”[9]及“1+15”政策体系[10]。成为全国科技进步考核先进市、国家知识产权试点城市、全国博士后创新示范中心试点和广东省小微企业创业创新综合改革试点市。江门中微子实验项目加快建设。全市高新技术企业数量增长2倍，总量达357家。新建3家国家级企业技术中心、2家院士工作站、115家省级工程技术研究中心。推动五邑大学开门办学，启动“环五邑大学创新经济圈”建设。江门火炬创业园、江门高新区成为国家级“小微双创”示范基地，推动“中国青创汇”、中国（江门）“侨梦苑”华侨华人创新产业聚集区、南方教育装备创新产业城等“双引双创”[11]平台落户，成功举办“中国青创汇”双创周活动。全市市场主体总数突破27万户，期末注册资本3465亿元，分别比2011年底增长42%、76%。

我们紧紧围绕供给侧结构性改革，制订有江门特色的“六新六去”[12]工作清单。成为“中国国际特色旅游目的地”创建城市和全省旅游综合改革示范市，全市旅游总收入连续五年增长超过20%，五年实现收入翻番。全面启动中国侨都潮人径建设，引进赤坎古镇华侨文化展示旅游项目、地中海俱乐部等旅游项目。金融生态环境不断优化，成为全国小微企业信用体系建设试验区，与澳门签订金融合作备忘录，新引入各类金融机构40家、总数达118家，境内外上市企业新增8家、累计达18家，新三板挂牌企业新增13家，完成广东省农村普惠金融建设恩平试点任务。加快发展现代农业，国家级农业主题公园、省级农产品加工示范园区、省农科院现代农业促进中心落户，五年来新增农民专业合作社1297家、市级以上重点农业龙头企业27家，“政银保”[13]工作经验在全国推广，建设高标准基本农田90.1万亩，获全省耕地保护考核一等奖。崖门国家一级渔港建成并投入使用。

过去五年，是交通突破、城局拓开的五年

我们主动谋划珠西综合交通枢纽江门站，由城际轨道支线变综合交通枢纽，并纳入省“十三五”规划；调整江门大道规划，由单“Y”型变双“Y”型，由交通干线变城市快速。开展交通大会战，五年累计交通建设投入超400亿元。建成江顺大桥、江门大道北、江门大道中五洞-东甲立交段主线、广珠铁路、江罗高速、广中江高速一期、新台高速南延线、佛开高速九江大桥至三堡段扩建等一批交通基础设施，江门大道南、台开快速路及中开、高恩等高速公路、深茂铁路、高新区码头等加快推进。“东部一体、西部协同”区域错位发展格局初步形成，中心城区框架进一步拉开，滨江新城加快建设，台开同城建设启动。

我们提出公园城市建设目标，创新设立公园建设分级标准，启动创建国家森林城市工作，打造七大龙头公园[14]，新增森林公园52个、湿地公园6个，新建改建村居公园773个，基本实现“一村（社区）一公园”。加大生态环境保护力度，通过立法形式加强对潭江流域、市区山体保护，实施河长制，城镇生活垃圾无害化处理率达100%、污水处理率达91%，空气质量总体居全国74个重点城市前列。节约集约用地水平不断提高，完成“三旧”改造[15]7034亩。

我们启动中国侨都“十个一”[16]建设，分别在马来西亚、香港和印尼成功举办三届世界江门青年大会，以“五邑银信”为重要组成部分的《侨批档案》入选世界记忆遗产名录，江门史迹点入选海上丝绸之路中国史迹首批申遗遗产点，开平塘口仓东村荣获联合国教科文组织亚太区文化遗产保护奖优秀奖。我们策划提出了五邑天地及蓬江33墟街、江海南岸印迹、新会学宫文心、鹤山岭南鹤武、台山海丝游归、开平碉楼逸风、恩平恩州学泉等特色文化品牌，启动第一批12个特色小镇[17]建设。

过去五年，是保障民生、共建共享的五年

我们统筹城乡精准扶贫精准脱贫，推进“大民政”工作，每年办好十大民生实事，加大公共服务投入，五年全市各级财政民生投入累计超700亿元，年均增长25%。全面实现贫困村基本脱贫，“邑家园”家庭综合服务中心实现村（居）全覆盖。实现企业职工养老、基本医疗、失业、工伤和生育保险市级统筹，实现城乡居民基本养老保险全覆盖、城乡居民基本医疗保险一体化，各项保障待遇稳步提升，低保标准、特困人员供养、孤儿供养、残疾人扶助等社会救助水平大幅提高。列入全国第一批医养结合试点地区，建立城乡一体化的居民社会养老保险制度。超额完成省下达的保障性住房建设和华侨农场、农村危房改造任务。解决8.5万农村居民饮水安全问题。大力促进就业创业，五年间全市城镇新增就业23.9万人，失业人员再就业16.6万人，建立省级高技能人才公共实训基地，每年新增高技能人才1万人以上。被授予“广东省推进教育现代化先进市”，所有市（区）通过国家义务教育发展基本均衡县验收。启动卫生强市建设，建成一批国家、省和市卫生镇村，基层医疗卫生服务体系和食品药品安全治理体系不断完善，“全面两孩”政策有序实施。建成江门演艺中心、生命安全体验馆和新的市工人文化宫、妇女儿童活动中心，江门体育中心建设扎实推进。荣获“中国舞蹈之城”称号，江门五邑联合文化场馆建设项目入选文化部国家文化创新工程，成功举办四届江门健走马拉松大赛。“平安江门”建设力度不断加大，成立食品药品与环境犯罪侦查支队。各类生产安全事故持续下降，社会大局和谐稳定。外事、港澳、对台、民族、宗教、人防、打私、优抚、双拥、统计、审计、新闻、出版、方志、气象、应急等工作均取得新进展。

过去五年，是简政提速、政府提效的五年

我们不断深化“三就”[18]，推行“两无两藏”[19]，持续推进“放管服”改革，多项措施走在全国、全省前列。出台涉企行政事业性、经营服务性、行政审批中介服务收费目录和“1+3”清单[20]，持续简政放权，五年市、县两级共调整审批事项3264项，精简比例达43%。完成新一轮政府职能转变和机构改革，理顺江门高新区、滨江新城体制机制，综合行政执法体制、简政强镇等改革稳步推进，推动镇（街）合并、园镇融合提质发展。探索推动以PPP模式[21]为重点的投融资体制改革，组建粤科江门创新创业投资母基金，成立江门市金融投资控股有限公司。大力推进商事制度改革，在全国率先实现工商登记“九证合一”[22]，在全省率先启用“双随机一公开”[23]抽查系统。落实“营改增”改革，实现国地税联合办税。建成跨境电子商务领域“单一窗口”。推动“互联网+政务服务”，开展“邑门式”行政服务[24]改革，自然人“邑门式”[25]实现镇（街）、村（居）全覆盖，法人“邑门式”[26]试点启动。开通“微市长”“邑网通”“邑微警”等网络服务平台，“微市长”获评“粤治”政府治理创新十佳案例。成立网络信息统筹局，建成覆盖全行业的政企通信息平台，开通公共资源电子交易全覆盖的“全市一张网”平台，获评全国十佳创新型公共资源交易机构。共办理人大代表建议586件、政协提案764件，满意率达99.1%和99.9%。被评为全国法治城市、法治县（市、区）创建活动先进单位、“六五”全国法治宣传教育先进城市，成为全省首批制定地方性法规的地级市，建立四级公共法律服务网络，一村（社区）一法律顾问工作实现全覆盖。扎实开展党的群众路线教育实践活动、“三严三实”[27]专题教育、“两学一做”[28]学习教育，落实中央八项规定精神，大力整治“四风”[29]，市直党政机关和参公单位“三公”经费[30]财政拨款总支出连续五年“零增长”。加强政府党风廉政建设，严格落实“一岗双责”，坚决查处违纪违法案件。

各位代表，过去五年我们在市委的正确领导下，在市人大、市政协与各民主党派的大力支持下，在全市人民、外来投资者和建设者以及驻江门军警部队的共同努力下，在港澳台同胞、海外侨胞以及国际友人的关心帮助下，经济社会发展建设都取得了显著进步。在此，向所有参与、支持和关心江门发展建设的各界人士，表示崇高的敬意和衷心的感谢！

我们也清醒地认识到江门发展仍存在短板和挑战：实体经济发展不快，自主创新能力不足，区域发展不平衡；环保问题依然突出，教育、医疗、卫生、养老、公交等公共服务供给不足，对困难群众和特殊群体的帮扶力度还需加大；政府债务风险防控压力大；公务员队伍敢闯会创、担当干事能力不足，执行力不强，政府廉政建设任务繁重。我们将采取更有力措施，认真加以解决。

今后五年的目标任务

今后五年政府工作的指导思想是：高举中国特色社会主义伟大旗帜，以邓小平理论、“三个代表”重要思想、科学发展观为指导，全面贯彻党的十八大和十八届三中、四中、五中、六中全会精神，深入学习贯彻习近平总书记系列重要讲话精神，按照“五位一体”[31]总体布局和“四个全面”[32]战略布局，坚持创新、协调、绿色、开放、共享的发展理念，围绕“三个定位、两个率先”[33]和“兴业惠民、治吏简政”的要求，坚持工业立市不动摇，落实“三战略、三带动”[34]，实施“三个千亿计划”行动[35]，深化供给侧结构性改革，创新加快发展，提升城市品质，增进民生福祉，加强政府建设，全力打造“三门”、建设“三心”，率先全面建成小康社会。

今后五年我市经济社会发展的主要目标是：经济综合实力显著增强，地区生产总值和人均地区生产总值增幅高于全国、全省和珠三角平均水平，经济质量明显提升，经济总量在全省位次前移，城乡居民收入与经济发展同步增长，产业发展迈向中高端水平，珠西综合交通枢纽格局初具雏形，重点领域和关键环节改革取得决定性成果，打造全省一流的政务环境、市场环境、社会环境，实现文明程度更高，城乡环境更美，社会治理更完善，人民有更多的获得感、幸福感，加快建设“珠西产城枢纽、文明岭南儒城、秀美五邑天地、世遗中国侨都”，到2018年与全省同步全面建成小康社会。

实现上述奋斗目标，要重点抓好六个方面工作：

一是坚持重微双擎，推动创新加快。建设“制造强市”，充分发挥重大平台作用，加快重大工程建设，推动重大项目突破，强化重大企业培育，继续擦亮“小微双创”品牌，着力以智造重振“江门制造”、以双创打造“江门创造”、以匠心夯实“江门质造”，改造提升旧动能，加快培育新动能，大力发展实体经济。集中力量实施“珠西战略”，提升产业平台发展水平，统筹抓好珠三角（江门）国家自主创新示范区、广东（江门）大广海湾经济区与中欧（江门）中小企业国际合作区三大平台建设，加快推动“1+6”核心园区扩能增效。围绕“4+1”产业体系[36]，加快发展新一代信息技术、高端制造、生物、绿色低碳、数字创意等战略性新兴产业，培育生产性服务业，做大做强轨道交通、重卡和商用车、新材料新能源及装备、教育装备、大健康等五大产业集群。扩大产业投资，持续保持先进（装备）制造投资增幅高于工业投资增幅、工业投资增幅高于固定资产投资增幅的态势。实施创新驱动发展战略，以高新技术企业培育为重点推动实现“五个倍增”[37]。坚持引进高层次人才和高技能人才并重，积极培育本地人才，打造人才强市。激发民营经济活力，打造全国“小微双创”之都、全球华侨华人双创之城，形成创新型经济发展格局。

二是坚持承东启西，推动区域协同。充分发挥江门区位优势，持续向东主动对接广佛都市圈、深港澳经济圈两大龙头，重点推动“珠中江阳”新型都市圈建设和“江深合作”“江佛一家”，主动融入粤港澳大湾区，并传递战略西拓能量，做好“传”“接”的“中卫”角色。实施大交通发展战略，加快打造珠西综合交通枢纽“一站四门户”[38]格局，重点建设客运、物流“两中心”，构建“三大黄金通道”[39]，打通深（中）江（阳）西拓大通道。推动构建集铁路、公路、机场、港口、城市轨道于一体的海陆空综合交通体系。进一步深化“东提西进、同城共融”战略，打造“一主一副”大城格局，建设“四宜”“五高”[40]的一流城市，促进“人产城”融合。东部蓬江新鹤作为主中心，以江门大道为主轴，稳妥推进行政区划调整，强化中心城区带动力与辐射力，打造“珠西新中心、深港澳基地”；西部台开恩以台开同城为牵引，以台开快速路建设为突破，构建江门“副中心”，带动台开恩协同共进，打造“江门西门户、粤西桥头堡”。

三是坚持青山绿水，推动环境优化。加强城市设计，推动城市修善，加快形成主体功能区布局。强化红线意识，严格落实生态控制线、林业生态红线、海洋生态红线、耕地保护红线，严格按照环境容量及总量控制目标进行结构调整和精准招商。持续绿化美化城市，推进公园城市建设，构建市域公园体系，创建国家森林城市。集中力量加强环境保护，推动生态修复，深入实施水、大气、土壤等污染防治行动，重点推进潭江流域、城市黑臭水体整治，消除劣V类水体。

四是坚持承古创今，推动文旅振兴。加强城市人文修育，培育文化整体意识，塑造城市精神，建设文化强市。守护城市历史文脉传承，以白沙文化为根源打造文明岭南儒城，加强世界遗产、名镇名村、历史建筑、历史街区和工业遗产等保护和活化，力争成为拥有三处世界遗产[41]的城市，打造“秀美五邑天地”文旅品牌。发挥“中国第一侨乡”优势，擦亮“中国侨都”城市品牌，建设侨务强市。巩固文明城市创建成果，提升城市文明水平与市民素质，全面构建现代公共文化服务体系，开展丰富多彩的普惠性文化活动，推动文化资源向基层延伸。壮大文化产业，促进文化与旅游、科技等融合发展。

五是坚持保障惠民，推动民生改善。持续加大民生投入，着力提升底线民生保障水平，实施就业创业优先战略，提高城乡居民收入水平，确保全面小康覆盖全市域的所有人口。从解决人民群众最关心最直接最现实的问题入手，促进教育资源优质均衡，补足区域性教育学位不足的短板，努力办市民满意的教育，推进“邑家园”和居家养老服务“两个全覆盖”，抓好六项底线民生保障[42]，全面完成精准扶贫精准脱贫任务，提高农村医疗服务水平，建立城乡医保一体化制度，加快公交一体，让人民群众共享更多的发展成果。

六是坚持去繁就简，推动政府提效。继续深化“三就”，推行“两无两藏”，深入推进现代市场体系、财政体制、投融资体制、行政管理体制等重点领域改革，打造市场化、国际化、法治化的营商环境，以全面深化改革之痛，解企业创业创新之锁，释社会市场活力之能，拓创新加快发展之路。坚持“市场事，市场定；社会事，社会建；政府事，公平办”。进一步明晰市县镇村权责利关系，市一级强化统筹协调监督，县一级突出建设管理服务，镇（街）、村（社区）重在社会治理和公共服务，促进上下联动、协调同步。

2017年重点工作

2017年是新一届政府承前启后的开局之年，也是率先全面建成小康社会的关键之年。今年政府工作的总体要求是全面贯彻落实党的十八大、十八届三中、四中、五中、六中全会、中央经济工作会议、省委十一届八次全会、省委经济工作会议和市第十三次党代会精神，围绕“兴业惠民、治吏简政”，把握稳中求进总基调，坚持发展第一要务，以“六新六去”全面推进供给侧结构性改革，紧盯实体经济，落实“三个千亿计划”行动，擦亮“小微双创”品牌，着力促发展、重创新、抓改革、优环境、惠民生、防风险，推动江门奋进前行。

今年全市经济发展的预期目标为生产总值增长7.5%以上，一般公共预算收入增长7.5%以上（其他指标见附件）。

做好全年工作，要围绕人产城融合，发展新经济、培育新动能，重点统筹推进产业振兴、创新驱动；大城格局、枢纽带动；实事惠民、服务联动；政府建设、改革推动。

一、产业振兴 创新驱动

重振“江门制造”。推动园区扩能增效，加快江门高新区华夏幸福珠西产业新城建设，启动龙溪湖水系核心区开发，打造全球华侨华人“双引双创”特色小镇；有序推动广东（江门）大广海湾经济区开发，优先发展先导区，启动粤澳产业合作示范区和珠西化工基地建设，争取台山核电投入商业运营；加快中欧（江门）中小企业国际合作区建设，完善核心区配套设施，加强与欧洲在机械装备、电力设备、电子信息、新能源汽车等产业对接合作。加大江沙示范园区建设力度，优先安排珠西智谷孵化成功企业落户。推进“1+6”核心园区建设，大力推动“七个一”专项行动[43]，盘活闲置土地1000亩以上、处置空置厂房10万平方米以上。推进江门信息大道建设，在全市域打造全光网城市，规划建设“数据工厂”，加快滨江新城省级大数据产业园建设。力抓工业投资，实施对工业投资特别是重大产业项目投资的专项考核。集中解决一批项目开工难、落地难问题，在用地、环保、建设等环节简化手续、提升效率，确保年内建成一批、新开工一批、引进一批优质产业项目。全面加大招商引资力度，建立全市招商统筹协调指导机制，做好产业规划和链条设计，集中精力集聚资源，落实以商引商、中介招商，推动精准招商，力求在重大产业项目引进和培育上取得新突破。鼓励企业增资扩产，实现“吹糠见米”。厚植民营经济，切实落实民营经济“十二条”[44]，让各项政策全部落地兑现，保障民营经济生存空间，提振民营企业发展信心，充分发挥各级工商联、商会、行业协会桥梁纽带作用，推动“邑商再出发”。市场主体净增2万家以上，力争总量突破30万家。加大骨干龙头企业培育力度，加快培育3家以上产值超百亿元、具有核心竞争力的龙头企业，培育更多在全国、全省同行业领先的“单打冠军”和“团体冠军”。弘扬“工匠精神”，促进品牌建设，重视老字号品牌的传承与保护，培育更多“百年老店”。推动外经贸持续正向增长，持续优化外贸结构，大力发展一般贸易，加快发展服务贸易，发展外贸新业态，加快搭建外贸综合服务平台，推动大广海湾保税物流中心（B型）申报，加大出口产品质量安全示范区建设力度，提高口岸通关效率，提升利用外资和外经贸效益水平。夯实“江门质造”，实施质量强市战略，启动“全国质量强市示范城市”“全国知名品牌示范区”创建工作，以打造国家地理标志、名牌产品、驰名（著名）商标和标准制修订为引领，创建广东省名牌产品15个，制修订国家标准、行业标准、广东省地方标准5项。提升现有检测服务平台能力，推动国家级珠西综合质量检测服务平台建设。

加快“江门创造”。抓好珠三角（江门）国家自主创新示范区建设，打造“1+6+N”创新格局[45]，围绕珠西智谷、珠西创城、冈州创客等创新载体，加快建设“四众”[46]平台，R&D[47]占GDP比重提升至2.3%以上。大力培育高新技术企业、引领型创新企业和科技型小微企业，高新技术企业数量突破500家，入库培育存量300家。精准扶持、重点培育科技型小微企业，总数突破2000家。推动科技成果实现产业化600项，创建50家企业工程技术研究中心，加大知识产权保护力度。加强引进各类新型研发机构，加快江门中微子实验项目进度，新建市级以上孵化器3家以上，组织200项市级重点科技计划项目，开展产学研项目100项。建设“环五邑大学创新经济圈”，打造开放式创新共同体。全力支持五邑大学加快创建特色鲜明广东省高水平工科大学，加强学科建设，加大各类高层次人才引进力度，成为高等人才培养的“蓄水池”。继续推动五邑大学开门办学，加强校企合作、校校合作、校政合作，推进五邑大学与各市（区）共建一批产业学院、研究院[48]，探索五邑大学与海外大学联合办学。打造“学院之城”，建立江门五邑高等学校（学院）校院联盟，支持江门职业技术学院建成省级示范性高等职业院校，并与台山合作办好新宁学院，力争设立广东江门中医药职业学院，加快推进广东江门幼儿师范高等专科学校建设，提升广东南方职业学院、江门技师学院、台山厨艺学院发展水平，推动江门市广播电视大学转型成为江门（五邑）开放大学，打造全民学习和终身教育的共享平台。巩固提升各市（区）中职学院发展，鼓励有条件的学校升格为高职院校。深化“小微双创”，抓好全国“小微双创”基地示范城市和省“小微双创”综合改革试点市建设，深入推进“中国青创汇”、中国（江门）“侨梦苑”华侨华人创新产业聚集区等建设，充分发挥“侨创基金”作用，完善提升小微企业技术、金融等公共服务平台。做好全国小微企业信用体系建设试验区工作，推动第三方信用评级。推进“小微双创”城市联盟建设，加强与广州、深圳、佛山等城市合作。创新人才培养、引进和使用制度，落实人才强市“十四条”[49]，完善人才评价机制，打造江门“人才岛”[50]。加强院士工作站、博士后科研工作站、博士后创新实践基地和五邑侨智库等新型智库建设，高标准建设全国博士后创新（江门）示范中心。开展企业家人才培训，培育“邑商精英”。探索建立中欧高技能人才培训合作（一试两证[51]）示范学院，建立高技能人才“菜单式”培训模式[52]，创建全国高技能人才示范城市。鼓励本地大学生回城返乡支持家乡建设。

推动“六新六去”。落实并完善供给侧结构性改革“六新六去”工作清单。加强对低效用地、低效厂房的盘活利用，重点围绕江门大道中加快一批“三旧”改造项目，实现“二次开发”和“二次利用”，鼓励“棚改厂”“工改工”“单层改多层”。健全新增建设用地指标分配与盘活存量建设用地挂钩机制，优先保障制造业项目用地。促进房地产去库存，研究建立房地产平稳健康发展长效机制。继续落实“六个优先”[53]，促进企业技术改造，完成技改投资增长25%以上，力促重大技改项目突破，推动“互联网+”、智能制造等新技术应用，继续在重点行业实施机器人应用工程，促进装备升级、工艺升级、产品升级。优化能源产业结构，深化电力体制改革。积极培育服务业新业态，加快发展工业设计、现代物流、电子商务、法律、中介等生产性服务业，整合设立江门“市长杯”工业设计大赛。以珠西会展中心建成运营为契机，促进全市会展资源与力量整合，发展壮大会展产业链，塑造会展品牌。探索现代商业模式。强化金融对企业的支撑，争取1－2家企业上市，鼓励中小微企业在新三板和股权交易中心挂牌融资，支持企业并购重组，做大做强现有上市公司。继续推动政府投融资改革，建立健全PPP项目管理运行机制，积极发展混合所有制经济。发挥先进装备制造产业基金（40亿元）、创业创新基金（20亿元）、PPP融资支持基金（50亿元）等政府投资基金的作用，支持实体经济与创新经济发展。完成国有“僵尸企业”出清重组工作，促进平台公司向实体化转型，持续推进国资优化重组，探索建立国资外派监事会制度，加快金控公司发展，推进国有资产资本化、证券化，推动国有企业重组上市。深化江澳金融合作。落实台山、开平普惠金融“村村通”建设。着力防风险，强化风险预警，应对财政收支等压力，促进经济健康平稳。

推进全域旅游。开展中国国际特色旅游目的地创建、世界旅游组织可持续发展中国观测点申报工作，构建环圭峰山国家旅游产业集聚区和台开恩世遗文化旅游区，推动台山和开平创建全国全域旅游示范区。推动旅游景区创A和龙头项目建设，加快碉楼文化旅游区创建国家5A级旅游景区工作，推进开平赤坎古镇项目建设，抓好新会美丽湖湾、台山滨海旅游、恩平航空小镇等龙头项目招商建设。按照“主客共享”全域旅游理念，加强游客集散中心、文旅步径、温泉旅居、旅游驿站、特色街区、特色民宿、游艇基地等基础工程建设，培育游艇、航空、工业、乡村等旅游新业态，推进“醉美江门百村”建设。

深化农业强市建设。大力发展现代农业，推进农业供给侧结构性改革，加大优势特色农产品产业扶持力度，积极培育新型农业经营主体、新型职业农民，加强与农业科研机构的合作，新增市级以上重点农业龙头企业10家以上、市级农民专业合作示范社15家、家庭农场15家以上、农业新型研发机构5家。继续推进农业“政银保”项目。抓好广东省农产品加工示范区、粤台（江门）农业合作试验区、以江门市现代农业综合示范基地为核心的“现代种业长廊”建设。全面建设镇级农产品质量监测站，创建国家农产品质量安全市。稳步推进第三次全国农业普查工作。加快推动气象现代化。落实粮食安全责任制。加快美丽乡村建设，推进新农村连片示范建设，新增开展73条行政村村庄整治，完成137条乡村绿化美化工程建设，加快推进台山全域开展农村人居环境综合整治。全面完成22.4万亩和启动新建20.2万亩高标准基本农田建设，加强水利设施和农村电网改造建设，提升“三防”应急能力。深入推进农村综合改革，深化新会全国农村综合改革示范试点成果，推进鹤山国家级扶持村集体经济发展试点工作，建设农村产权流转管理服务平台，完善农村集体“三资”[54]管理。推进农村土地承包经营权确权，年底完成颁证超过70%。推进“政经分开”改革，探索农村土地所有权、承包权、经营权“三权分置”，进一步强化基层党组织领导，明晰自治组织、经济组织和社会组织的权责利，重点理顺好农民、村（居）民、社员股民的权益关系。大力发展海洋经济，合理开发海洋资源，开展第一次全国海洋经济调查。加快渔船更新改造，发展远海捕捞业，推进台山烽火角区域性避风锚地、下川岛独湾码头防波堤建设。

二、大城格局 枢纽带动

深化“东提西进”。构建“一主一副”城市空间布局，蓬江重点谋划滨江新城智慧城，打造金融商务会展中心和总部经济区，建设人产城融合发展的现代生态新城；江海重点谋划科创新城，聚合全市创新资源，推动高新区在全国位次前移；新会重点谋划枢纽新城，以珠西综合交通枢纽江门站建设重振门户功能，并推动会城构造“一轴三心”[55]格局；鹤山重点谋划物流新城和鹤山工业城，加快建设珠西物流枢纽中心、装备制造业重点发展区。扎实推进台开恩协同特别是台开同城交通工程、公交一体、产业园区、环境治理、服务窗口等一批示范工程建设，支持台山、开平率先实现服务事项全城通办，提升台开同城人居环境；台山主动谋划大广海湾“三港融合”（海港、铁路港、空港），重点发展台山工业新城，建设国家新型城镇化标准化试点，强化与开平的同城对接；开平重点提升翠山湖科技产业园建设，并加强沿潭江“三江六岸”[56]的规划，激活旧城；恩平重点谋划“一园三区”[57]联动发展，主动融入台开同城，提升在西部台开恩协同中的地位。提升城乡规划建设管理水平，结合新一轮城市总体规划、土地利用总体规划编制及城市规划体系完善，加强对重点发展区域的规划控制。加大控制性详细规划编制力度，全面落实“一张图”规划、“一个系统”管理、“一套标准”审批的规划管理模式。加大违法用地、违章建筑整治力度。加快城市管理体制改革，明确蓬江、江海建设管理主体责任，推进城市管理和综合执法数字化、精细化、智慧化，开展不动产登记数据整合和登记信息管理基础平台建设。逐步建立城乡统一的新型户籍制度，提高户籍人口城镇化率。加快推进地下综合管廊及“海绵城市”[58]建设工作。

推进“交通会战”。打造珠西综合交通枢纽“一站四门户”格局。全力加快珠西综合交通枢纽江门站建设，推动站房同步合建，力争于2018年6月与深茂铁路江茂段同步建成投入使用，推进广珠铁路客运改造工程动工准备，启动广佛江珠城轨建设，加紧深茂铁路深圳至江门段、江肇铁路前期工作。推进江门北站（鹤山）规划建设，加快南沙港铁路建设和鹤台铁路前期规划工作，打造广佛物流集散地。提升江门东站（江海）建设运营水平，谋划江门西站（台开恩）、江门南站（台山广海湾）。加快高速快速道路建设，江门大道南（东线）建设实现银鹭－三江段主道建成通车，三江－金门段上半年动工，江门大道北、江门大道中五洞-五邑路段辅道工程建成，迎宾西路江门大道中－杜阮互通段通车，抓好会港大道、五邑路的快速化改造，规划建设东部蓬江新鹤快速路网；加快台开快速路工程建设，推动国道G325改线（台开恩联城快速）、国道G240改线前期工作，打造西部台开恩联城路网；中开高速江门段率先动工建设，加快银洲湖大桥及双水至水步段建设；广中江高速二期建成通车，加快高恩高速、开平至阳春高速建设和沈海高速三堡至水口段、开阳段扩建，开展新会至高栏港、开平至台山、珠海香洲至台山、斗门至恩平等高速前期工作。推进完善综合交通体系。加快编制《江门市综合交通一体化规划（2016－2030）》。启动通用航空机场规划建设，积极谋划对接珠三角新干线机场（广州二机场）。加快高新区码头建设，开展银洲湖、广海湾港区规划建设，推动那扶河及镇海湾出海航道疏浚工程。加快提升公交一体化水平，整合提升全市汽运资源，重点推进市域公交化，利用江门大道率先实现东部快速公交，推进公交候车亭和首末站场建设改造，启动城市轨道交通前期研究工作。

建设公园城市。推进一批国家级、省级公园建设，提升圭峰山、白水带、龙舟山、大雁山、石花山、梁金山、鳌峰山等龙头公园建设水平，推进新会小鸟天堂、开平孔雀湖国家湿地公园建设，规划建设蓬江湖水系、江海礼乐田园、新会思成湖水系、鹤山古劳水乡、台山大隆洞、恩平沙湖等湿地公园，基本建成台山中国农业公园，广东镇海湾红树林湿地公园形成雏形，完成江门市植物园兰石珍稀植物园区及绿护桃源景区建设。新建改建145个村居公园，新增10个森林公园，建成5个镇级公园，新建慢行系统和城乡绿廊60公里。抓好创建国家森林城市工作，开展多种形式、多元投入的植树造林活动，加强林业生态重点工程建设与管护工作，全力推进生态公益林扩面和饮用水源水库第一重山商品林调整，省级以上生态公益林面积占林业用地比重提升至37%。强化森林防火基础设施建设。

加强环境整治。全面落实环保规划和环境功能区划，严格执行生态分区管理要求，坚守饮用水源保护区、自然保护区和生态严控区等环保“红线”，严把建设项目环保准入关。落实珠三角建设国家绿色发展示范区工作，开展生态文明示范建设。加大水环境综合整治力度，强化饮用水源保护，深入落实河长制，实施挂牌督办，重点抓好“一面六河”（潭江牛湾断面和天沙河、杜阮河、麻园河、龙溪河、会城河、紫水河6条重度黑臭水体）污水综合整治。加强工业污染源、农业污染源、生活污水和垃圾等专项整治，重点推进畜禽养殖业污染治理。加快新一轮镇村污水处理设施建设，完成恩平建设示范县任务，全市城镇污水处理率达到92.5%。完善城乡生活垃圾收运体系，推进城乡生活垃圾资源化利用和分类减量处理，全市农村生活垃圾有效处理率达98%以上。加强环境监管执法，严厉打击废水废气偷排等环境违法行为，落实去污染工作任务。加强机动车污染防治和城市扬尘污染防控，推进文明施工，确保空气质量稳定达标并持续向好。推进全市皮革、印染、陶瓷等重点行业和蓬江荷塘、台山广海、恩平沙湖等重点区域的环保专项整治工作。加强重金属、土壤、固体废物污染防治。

建设“世遗中国侨都”。坚持以“侨”为“桥”，着力聚侨情、引侨资、借侨智、重侨创。办好第三届世界广府人恳亲大会暨2017中国侨都（江门）华人嘉年华活动。加强江港澳合作，谋划融入粤港澳大湾区规划建设，加强与“一带一路”[59]沿线国家和地区合作，完善全球华侨华人“邑门式”服务平台。推进海上丝绸之路史迹点申遗，完成遗产点环境整治和保护展示工程，创建省级华侨文化生态保护实验区。建设“秀美五邑天地”，统筹整合蓬江33墟街、江海南岸印迹、新会学宫文心、鹤山岭南鹤武、台山海丝游归、开平碉楼逸风与恩平恩州学泉，推动第一批12个特色小镇建设，建成南粤古驿道江门示范段（海口埠－梅家大院）。继续推进自然村落历史人文普查工作。整合提升公共文化服务，提高江门五邑联合图书馆、文化馆、博物馆、美术馆综合服务水平，加快推进文化馆、美术馆建设和华博馆、图书馆升级改造。搭建“江门文化云”，实现市县两级公共图书馆通借通还，与镇（街）、村（居）、企业、中小学校和各高校图书馆资源共享，推进全民阅读。传承传统文化，提升江门五邑粤剧曲艺联盟影响力，进一步打响侨乡武术文化品牌。推进江门市档案中心建设和各市（区）国家综合档案馆达标工作。加大对公共文化的政府购买服务力度。推动传媒资源整合。加快工艺美术、印刷发行、健身竞技等文化体育产业发展。

三、实事惠民 服务联动

加大民生投入，全市安排民生支出227.2亿元，同比增长15%。进一步巩固创文成果，做好全国文明城市复评工作，按照制度统一、标准一致、水平均衡的原则，深入推进基本公共服务均等化综合改革，抓好十件民生实事落实，深化“大民政”工作，让全市人民共享改革发展成果。

坚持教育优先。加强学校规划建设，落实义务教育阶段公办学校建设“三二一”工程[60]，全市计划启动建设17所学校。推进学前教育优质普惠发展，进一步完善住宅小区配套幼儿园管理，全市规范化幼儿园比例达80%以上。完善教师队伍交流轮岗制度，促进义务教育优质均衡。推动普通高中教育优质特色多样化发展。继续推进国家级特殊教育改革试验区建设。创建广东省职业教育综合改革示范市，深化产教融合，推进现代职业教育结构优化，鼓励实践现代学徒制。大力扶持民办教育规范特色优质发展，支持混合所有制办学等新模式。进一步发展特色社区教育、老人教育，以江门社区大学、各市（区）社区学院为主体，整合社会各类教育资源。大力推进教育“名师工程”，加强高素质专业化教师队伍建设。加快江门智慧教育云平台建设，推进教育信息化。

建设“健康江门”。深化医药卫生体制改革，创建全省深化医药卫生体制综合改革示范市。提升县级公立医院医疗服务能力，争取县域内就诊率达到90%左右，基本实现大病不出县。开展“医联体”[61]管理，力争10月前完成县镇村医疗卫生服务一体化管理，完成10家基层医院的镇（街）分院建设、16家乡镇卫生院标准化建设和125间村卫生站公建民营建设，完善农村医疗服务。加快全市区域卫生信息平台建设，探索“互联网+分级诊疗”模式。加大对中医药医疗行业人才培养力度。加快发展社会办医，鼓励社会资本开办医疗机构。全面深化爱国卫生运动，积极探索健康城市、健康村镇建设，加强重大疾病防控，提升卫生应急能力。大力推广家庭医生制度、强化付费方式改革等医改举措。进一步完善食品药品监管体系建设，启动创建食品安全示范城市。加强计生服务管理，促进人口长期均衡发展。完善公共体育服务体系，建成并开放江门体育中心，加强社区体育设施建设。

推动精准扶贫。继续探索和提升统筹城乡精准扶贫精准脱贫工作，构建常态化、全覆盖的城乡扶贫长效机制，推动扶贫与低保“两线合一”，确保贫困发生率控制3%以内，实现30%以上建档立卡贫困户共5000人精准脱贫。继续开展百企扶百村、百医牵百村、千（万）义工助千户行动等不同类型社会扶贫模式，支持慈善机构参与扶贫开发。建立稳定的村级公共服务管理和资源管护考核奖补机制，村委会年集体收入进一步提高至18万元以上。

提升底线保障。不断提高城乡最低生活保障、特困人员供养、孤儿供养、城乡医疗救助、困难残疾人生活补贴和重度残疾人护理补贴标准，筑牢政府底线保障网。完善重特大疾病医疗救助制度，提高医疗救助标准，普及医疗救助和医疗保险同步结算服务。逐步建立适度普惠型儿童福利体系，加强关爱留守儿童工作。进一步完善残疾人社区康园网络建设，每个镇（街）至少建立1个社区康园中心。扶持380户困难残疾人家庭发展生产和自主创业。强化住房保障，完成棚户区和农村危房改造任务。

促进服务均等。统筹推进“邑家园”与农村基层公共服务平台建设，将各镇（街）“邑门式”与枢纽型“邑家园”有机结合，使“邑家园”成为“邑门式”的服务终端，推动服务延伸至村（居），实现服务就在“家门口”。推动“邑家园”+社区教育、“邑家园”+志愿服务，构建“三建”（党建社建群建）齐动、“三社”（社区社组社工）联动、“三工”（党工社工义工）互动基层服务模式，以普及电子义工证为纽带，以各村（居）“邑家园”全面建立志愿服务站为平台，以基层党员志愿服务带动社会志愿服务。建设以居家为基础、社区为依托、机构为支撑的养老服务体系，新增养老床位1500张，建立居家养老服务设施长效运作机制。引导社会组织规范有序发展并参与基层社会治理。

完善就业保障。实施就业实名制，开展精准化就业帮扶，鼓励多渠道多形式就业，全市城镇新增就业4.5万人以上，城镇失业人员再就业3万人，应届高校毕业生就业率达90%以上。加强本地紧缺专业人才培养，实施全民技能提升计划。完善就业创业政策，推进创业孵化基地建设，树立创业导师品牌。积极创建和谐劳动关系。适当降低社会保险费率，推进社会保险全覆盖，提高社会保险待遇，加强社保基金监管。完善全征地农民养老保障政策和社保基金预警机制。推进城乡医疗保险一体化。拓展社会保障卡功能。

打造平安江门。完善立体化、信息化社会治安防控体系，推进“综治中心+网格化+信息化”工作，开展“雪亮工程”[62]，深化平安创建。推进智能感知采集网、社会治安视频监控系统和云端警务建设，全面推广应用“门禁+视频”系统，打造“江门邑微警”网上公安局。树立安全发展理念，强化城市安全意识。实行维稳综治及安全生产党政同责、“一岗双责”、齐抓共管、失职追责。公开安全“黑名单”，抓好重点领域安全隐患排查整治，深入推进城市风险点、危险源排查管控，坚决遏制较大以上事故发生。进一步完善“大调解”工作格局，建立健全社会矛盾纠纷预防化解机制。完善突发事件应急机制建设，提高应急管理能力。

此外，进一步完善国防动员体系，落实双拥和优抚安置工作。继续做好统战、民族、宗教、对台、人防、打私、统计、方志、妇女儿童、红十字会等工作。

四、政府建设 改革推动

服务简便高效。要大行简政之道，深化以“三制”[63]为重点的“放管服”改革，“放”要快、“管”要精、“服”要优，营造一流的政务环境，进一步激发社会创造活力。加快简政放权。坚持简政先减证，按能放尽放、应放快放原则，放宽市场准入，降低准入准营门槛。深化商事制度改革，一季度实现银行开户许可等更多证照纳入“多证合一”改革，形成较为完整的市场准入便利化链条。积极推进电子营业执照和全程电子化登记，探索推动“证照分离”。实施承诺制，争取上半年实现政府投资项目试行承诺制，企业投资项目在“1+6”园区试点承诺制，加快项目落地。推行委托制，加快推进对“1+6”园区的委托授权，分期分批赋予“1+6”园区市一级经济管理权限。实施精准监管。全面推进“双随机一公开”监管，力争实现市县两级所有执法事项全覆盖，探索联合执法抽查，加强事中事后监管。深化清单制，加快推进行政审批事项标准化工作，抓好“1+3”清单动态管理。推动省以下环保机构监测监察执法垂直管理制度、审计制度、城市执法体制、盐业体制等改革。建立健全跨部门协同监管和联合惩戒机制，推进社会诚信体系建设。优化政府服务。按照标准、规范、透明的原则，推进“互联网+政务服务”，加快政务数据资源汇聚统筹，提升“12345”政府服务热线水平，完善“邑网通”“微市长”等政务服务平台，上半年完成法人“邑门式”行政服务改革和公共服务标准化建设，实行行政审批事项标准线上线下一体化运行，宁可数据在网上多跑几圈，也要让企业和群众少跑一轮。

办事依法透明。推进依法治市，建设法治政府和诚信政府。用好地方立法权，推进城乡建设与管理、环境保护、历史文化保护等领域立法。主动接受人大监督，认真执行市人大及其常委会各项决议决定，支持政协委员参政议政，提高人大代表议案、建议和政协提案办理水平。完善重大行政决策民主化、科学化、法治化程序。继续推进“七五”普法工作，开展高标准法治创建活动，全市60%以上镇（街）创建法治镇（街）。加强一村（社区）一法律顾问制度，完善公共法律服务实体平台建设。推进“两建”[64]工作，营造法治化营商环境。加强政务公开。完善财政预算执行管理，建立健全市县两级财政支出进度通报制度，落实支出进度与预算安排挂钩机制。建立市直单位网上办事大厅行政审批绩效评价机制，推进第三方评估行政审批和政务服务效能。探索建立行政执法裁量权管理及执法监督网上系统。继续推进公共资源交易体制改革，整合建立统一的公共资源交易平台，开展电子监管和信用管理，积极推进全省异地评标试点工作，探索公共资源大数据监测及分析。

队伍担当作为。抓好廉政建设，完善监察、司法、审计等联动的监督机制，严肃查处各类违纪违法案件。坚持厉行节约，强化“三公”经费管理，对公共资金、国有资产、国有资源和领导干部履行经济责任情况实行审计全覆盖。全面推行无纸化办公。健全问责机制，强化绩效管理，严肃整治行政“不作为、乱作为、慢作为”现象，锤炼一支不等不靠、不推不让、干净干事的公务员队伍。切实提升执行力，着力解决“一停二看三犹豫”问题，绝不允许以吃瓜群众的心态玩套路，必须敢闯会创、率先行动，以洪荒之力推动工作落实，结果说话。

各位代表，同志们，“鸿鹄寄云霞，奋进趁年华”。未来五年是江门推动“兴业惠民、治吏简政”的关键五年，任务光荣而艰巨，我们不做跟跑者、要做领头羊；不做守成者，要做创新客。新一届政府将在市委的正确领导下，不忘初心、继续前进，不辜负全市人民和所有关心江门发展建设的各界人士的殷切期望，齐心协力把江门发展好、建设好，率先全面建成小康社会，共创幸福美好生活！
